# Inflammatory cytokines and immune system modulation by aerobic versus resisted exercise training for elderly

**DOI:** 10.4314/ahs.v18i1.16

**Published:** 2018-03

**Authors:** Shehab M Abd El-Kader, Fadwa M Al-Shreef

**Affiliations:** 1 Department of Physical Therapy, Faculty of Applied Medical Sciences, King Abdulaziz University, Jeddah, Saudi Arabia; 2 Department of Medical Laboratory Technology, Faculty of Applied Medical Sciences, King Abdulaziz University, Jeddah, Saudi Arabia

**Keywords:** Immune function, inflammatory cytokines, aerobic exercise, resistance exercise, aging

## Abstract

**Background:**

Aging is characterized with immunosenescence associated with a hyper-inflammatory state, characterized by elevated circulating levels of pro-inflammatory mediators. Physical exercise is a potential strategy for improving the immune system dysfunction and chronic inflammation that accompanies aging. However, there is a need to differentiate between aerobic and resistance exercise training regarding human immune system and systemic inflammation among the elderly Saudi population.

**Objective:**

The aim of this study was to compare the impact of 6 months of aerobic versus resisted exercise training on inflammatory cytokines and immune system response among elderly.

**Material and methods:**

Sixty previously sedentary elderly subjects participated in this study, their age ranged from 61–66 years. All Subjects were randomly assigned to supervised aerobic exercise intervention group (group A, n=40) or resistance exercise group (group B, n=40). Number of CD3^+^,CD4^+^,CD8^+^ T cells count and CD4/CD8 ratio were quantified, IL-6, TNF-α and IL10 were measured before and after 6 months, at the end of the study.

**Results:**

The mean values of CD3^+^, CD4^+^ and CD8^+^ T cells count and IL-10 were significantly increased, whereas the mean values of CD4/CD8 ratio, IL-6 and TNF-α were significantly decreased in group (A) and group (B). Also; there were significant differences between mean levels of the investigated parameters in group (A) and group (B) after treatment.

**Conclusion:**

The current study provides evidence that aerobic exercise is more appropriate in modulating the immune system and inflammatory markers among the elderly population.

## Introduction

Human aging is associated with a progressive decline in the function of the immune system, which is commonly referred to as immunosenescence. The adaptive arm of the immune system (i.e., T-cells, B-cells and their products) appears to diminish most with increasing age^1^. This is characterized by poor vaccine efficacy, increased incidence of opportunistic infections, and high morbidity and mortality among the elderly^2^.

Inflamm-aging was initially described as an increase in circulating concentrations of classically pro-inflammatory cytokines. However, there are many complex alterations in adaptive and innate immunity that also influence the secretion of anti-inflammatory and pro-resolving cytokines^3^. With regard to systemic inflammation, elevated circulating levels of the acute phase protein C-reactive protein (CRP) and cytokines, such as tumor necrosis factor alpha (TNF-α) and interleukin 6 (IL-6), have been found in elderly individuals^4^. Inflammation is considered to play a role in disease development and prognosis^5^, and high levels of inflammatory markers are associated with an increased risk of development of cardiovascular disease and cancer^6^,^7^.

Regular exercise is associated with decreased incidence of different types of chronic diseases. Part of the protective effect of exercise is related to changes in immune function, which may improve various aspects of wound healing, including macrophage polarization and functional status. Regular exercise has also been documented to be associated with reduced cancer risk and delayed tumor progression^8^. Exercise training interventions in previously sedentary elderly individuals have been shown to enhance T-cell proliferative capacity^9^,^10^.

Physical exercise is effective in reducing (or ameliorate) the risk of age-associated diseases. In fact, there is evidence supporting the involvement of inflammatory mechanisms with the beneficial effects of physical exercise, such as decrease in age-associated immune senescence^11^, increase in innate immune function^12^ and decrease in chronic inflammation^13^. It has also been reported that exercise training/physical activity are able to modulate the circulating levels of not only frequently measured cytokines such as IL-6 and TNF-α, but also other less frequently investigated cytokines^14^–^16^.

Aerobic exercise has been largely employed, but more recently, resistance exercise has been suggested, especially for the elderly population, because of its better effect on the functional capacity to perform activities of daily living regardless of health status^17^,^18^. Aerobic and resistance exercise training has been recommended as an anti-inflammatory therapy^19^–^21^. Subsequently, aerobic and resistance exercise have also been suggested to counter immunosenescence^22^–^24^. some research on aerobic exercise training has suggested improvements in the immune system for elderly subjects^25^–^27^ and others find effects of resistance training on the immune parameters of healthy elderly subjects^28^,^29^. However, published data is still controversial as resistance exercise training has failed to affect immune function in the elderly^20^,^30^.

As there is limitation in studies reporting the differences between the benefits of aerobic and resistance exercises on immune system response and systemic inflammation among elderly Saudi subjects. The aim of this study was to compare the impact of 6 months of aerobic versus resisted exercise training on inflammatory cytokines and immune system response among Saudi elderly.

## Patients and methods

### Subjects

**Figure (1):**
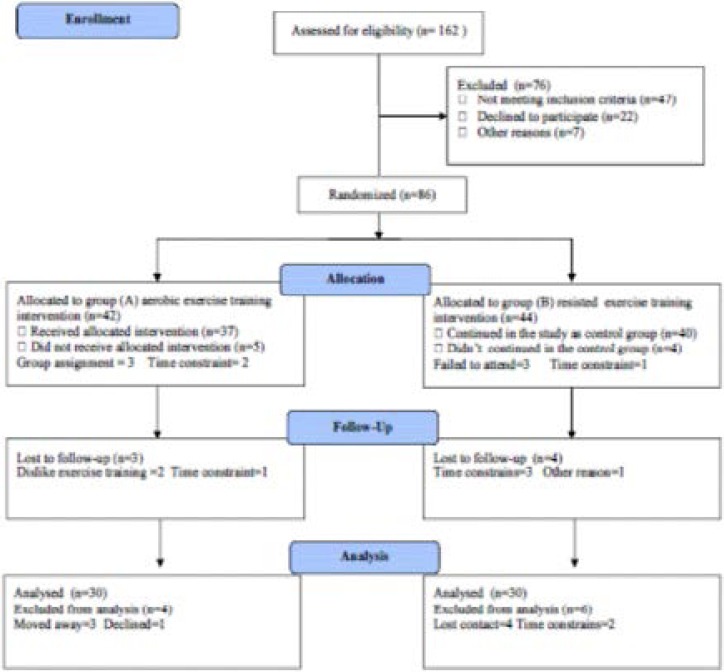
Subjects screening and recruitment CONSORT diagram.

Sixty sedentary Saudi elderly volunteers; their age ranged from 61 to 67 years, recruited from the community. They were considered sedentary if they had not performed exercise of 15 minutes duration more than 2 times per week for the previous 6 months. Subjects were excluded if they smoked, if they were taking any medications (i.e. aspirin, anti-inflammatory drugs, anti-depressants) known to affect immune function, if they had any recent (less than 3 months) surgery, infection, or vaccination, or if they reported a previous history of cancer, arthritis, or immune disorders. All subjects were cleared for participation by their personal physician, reported willingness to be randomly assigned to treatment conditions, and agreed not to participate in exercise outside the study. No attempts were made to control dietary intake. Subjects were randomized to either an aerobic exercise intervention group (group A) or resistance exercise intervention group (group B). Both groups participated in the exercise intervention conducted 3 times per week for 6 months (starting in April–June and ending in October–December). Exercise sessions were supervised and monitored by trained exercise specialists. The CONSORT diagram outlining the details of the screening, run-in and randomization phases of the study and reasons for participant exclusion is illustrated in figure (1). Informed consent was obtained from all participants. This study was approved by the Scientific Research Ethical Committee, Faculty of Applied Medical Sciences at King Abdulaziz University.

### Measurements

Laboratory analysis was performed by independent assessors who were blinded to group assignment and not involved in the routine treatment of the patients, however the following measurements were taken before the study and after 6 months at the end of the study:

**A. Inflammatory cytokines:** Blood samples were drained from the antecubital vein after a 12-hour fasting, the blood samples were centrifuged at + 4 °C (1000 = g for 10 min). Interleukin-6 (IL-6) and Interleukin-10(IL-10) levels were analyzed by “Immulite 2000” immunassay analyzer (Siemens Healthcare Diagnostics, Deerfield, USA). However, tumor necrosis factor-alpha (TNF-α) was measured by ELISA kits (ELX 50) in addition to ELISA microplate reader (ELX 808; BioTek Instruments, USA).

**B. Flow cytometry analysis:** The human leukocyte differentiation antigens CD3, CD4 and CD8 (Beckman Coulter, Marseille, France) Five microliters of appropriate monoclonal antibody was added to 50 µL of a wholeblood sample and incubated for 15 minutes at room temperature. Thereafter, the erythrocytes were lysed with 125 µL of a lysing solution, OptiLyse C, for 10 minutes. The reaction was stopped by the addition of 250 µL phosphate-buffered saline. The samples were analyzed by flow cytometry using Cytomics FC 500 and CXP software (Beckman Coulter).The leukocyte subsets were defined by forward- and side-scatter pattern. The negative control value was determined by a fluorescence background and antibody-nonspecific staining.

### Procedures

**A. Aerobic exercise training program:** Patients in group (A) were submitted to a 40 min aerobic session on a treadmill (the initial, 5-minute warm-up phase performed on the treadmill at a low load, each training session lasted 30 minutes and ended with 5-minute recovery and relaxation phase) either walking or running, based on heart rate, until the target heart rate was reached, according to American College of Sport Medicine guidelines. The program began with 10 min of stretching and was conducted using the maximal heart rate index (HRmax) estimated by: 220-age. First 3 months = 60–70% of HRmax, second 3 months = 70–80% of HR_max_^31^.

**B. Resistance exercise training:** Patients in group (B) were submitted to a 40 min session of resistance training. The program began with 10 min of stretching and was conducted with exercises done on nine resistance machines. The resistance machines used were: chest press, bicep curl, triceps extension, lower back, abdominals, leg press, leg curl and leg extension. Subjects performed three sets of 8–12 repetitions, with 60 s of rest between each set. Resistance was increased by five pounds after the subject was able to complete three sets of eight repetitions on three consecutive days. Subjects were trained using between 60 and 80% of their one maximal repetition weight (1-RM)^32^.

### Statistical analysis

The mean values of the investigated parameters obtained before and after three months in both groups were compared using paired “t” test. Independent “t” test was used for the comparison between the two groups (P<0.05).

## Results

The two groups were considered homogeneous regarding the demographic variables (table 1). The mean age of the group (A) was 66.43 ± 3.71years, and the mean age of group (B) was 65.96 ± 3.42 years. There were no significant differences in age, weight, height, body mass index (BMI), systolic blood pressure, diastolic blood pressure, and maximal heart rate (HRmax) between both groups.

**Table 1:** Baseline characteristics of study participants.

Characteristic	Group (A)	Group (B)	Significance
**Age** (years)	66.43 ± 3.71	65. 96 ±3.42	P>0.05
**Weight** (kg)	71.15 ± 6.28	68.22 ± 7.53	P>0.05
**Height** (m)	1.67± 0.08	1.70 ± 0.07	P>0.05
**BMI** (kg/m^2^)	24.13 ± 3.64	23.21 ± 3.12	P>0.05
**SBP** (mmHg)	131.61 ± 9.27	129.45±10.33	P>0.05
**DBP** (mmHg)	84.22 ± 4.31	83.31 ± 4.61	P>0.05
**HR_max_** (beat/min)	155.58 ± 10.25	152.91 ± 11.47	P>0.05

BMI: Body mass index; SBP: Systolic blood pressure; DBP: Diastolic blood pressure; HR_max_: Maximum heart rate.

There was a 32.7%, 31.8%, 32.1%, 21.9%, 33.7% and 24.3% reduction in mean values of TNF-α, IL-6, CD3 count, CD4 count, CD8 count and CD4/CD8 ratio respectively in addition to 28.4% increase in IL-10 of the training group (table 2). While, there was a 3.5%, 3.3%, 4.9%, 2.9%, 3.7% and 3.4% increase in mean values of the same variables and 3.8% increase in IL-10 in the control group. The mean values of TNF-α, IL-6, CD3 count, CD4 count, CD8 count and CD4/CD8 ratio decreased significantly in addition to significant increase in the mean value of the IL-10 in the training group, however the results of the control group were not significant (table 3). Also, there were significant differences between both groups at the end of the study (table 4).

**Table 2:** Mean value and significance of TNF-α, IL-6, IL-10, CD3 count, CD4 count, CD8 count and CD4/CD8 ratio in group (A) before and at the end of the study.

	Mean + SD		t-value	Significance
	Pre	Post		
**TNF-α** (pg/mL)	4.77 ± 1.62*	3.21 ± 1.34	6.27	P <0.05
**IL-6** (pg/mL)	2.58 ± 0.93*	1.76 ± 0.81	5.45	P <0.05
**IL-10** (pg/ml)	5.94 ± 1.25	7.63 ± 1.32	6.91	P <0.05
**CD3 cell** **count** (10^9^/L)	1.93 ± 0.87*	1.31 ± 0.75	5.24	P<0.05
**CD4 cell** **count** (10^9^/L)	1.41 ± 0.92*	1.10 ± 0.78	6.15	P <0.05
**CD8 cell** **count** (10^9^/L)	0.86 ± 0.33*	0.57 ± 0.26	5.14	P <0.05
**CD4/CD8 ratio**	1.52 ± 0.86*	1.15 ± 0.71	5.23	P <0.05

TNF-α: tumor necrosis factor — alpha; IL-6: Interleukin-6; IL-10: Interleukin-10;

(*) indicates a significant difference between the two groups, P < 0.05.

**Table 3:** Mean value and significance of TNF-α, IL-6, IL-10, CD3 count, CD4 count, CD8 count and CD4/CD8 ratio in group (B) before and at the end of the study.

	Mean + SD		t-value	Significance
	Pre	Post		
**TNF-α** (pg/mL)	4.56 ± 1.43	4.72 ± 1.57	0.94	P>0.05
**IL-6** (pg/mL)	2.40 ± 0.87	2.48 ± 0.92	0.76	P>0.05
**IL-10** (pg/ml)	6.36 ± 1.43	6.19 ± 1.36	1.12	P>0.05
**CD3** **count** (10^9^/L)	1.82 ± 0.85	1.91 ± 0.86	0.82	P>0.05
**CD4** **count** (10^9^/L)	1.35 ± 0.84	1.39 ± 0.87	0.65	P>0.05
**CD8** **count** (10^9^/L)	0.81 ± 0.30	0.84 ± 0.33	0.71	P>0.05
**CD4/CD8 ratio**	1.48 ± 0.91	1.53 ± 0.86	0.88	P>0.05

TNF-α: tumor necrosis factor — alpha; IL-6: Interleukin-6; IL-10: Interleukin-10.

**Table 4:** Mean value and significance of TNF-α, IL-6, IL-10, CD3 count, CD4 count, CD8 count and CD4/CD8 ratio in group (A) and group (B) at the end of the study.

	Mean + SD		t-value	Significance
	Group (A)	Group (B)		
**TNF-α** (pg/mL)	4.56 ± 1.43*	4.72 ± 1.57	6.71	P <0.05
**IL-6** (pg/mL)	2.40 ± 0.87*	2.48 ± 0.92	5.80	P <0.05
**IL-10** (pg/ml)	6.36 ± 1.43*	6.19 ± 1.36	7.23	P <0.05
**CD3** **count** (10^9^/L)	1.82 ± 0.85*	1.91 ± 0.86	5.82	P <0.05
**CD4** **count** (10^9^/L)	1.35 ± 0.84*	1.39 ± 0.87	6.77	P <0.05
**CD8** **count** (10^9^/L)	0.81 ± 0.30*	0.84 ± 0.33	5.61	P <0.05
**CD4/CD8 ratio**	1.48 ± 0.91*	1.53 ± 0.86	5.74	P <0.05

TNF-α: tumor necrosis factor — alpha; IL-6: Interleukin-6; IL-10: Interleukin-10;

(*) indicates a significant difference between the two groups, P < 0.05.

## Discussion

With aging, the immune system undergoes a remodeling process termed immunosenescence^33^. There is good evidence corroborating the use of exercise as a strategy to ameliorate physiological age-associated changes as well as an adjuvant strategy in the disease therapy^34^. In general, aerobic exercise has been largely employed, but more recently, resistance exercise has been suggested, especially for the elderly population, because of its better effect on the functional capacity to perform activities of daily living regardless of health status^35^,^36^. Subsequently, aerobic and resistance exercise have also been suggested to counter immunosenescence^37^,^38^.

To the best of our knowledge, this is the first comparative study between aerobic and resistance exercises addressing inflammatory and immunological parameters among elderly subjects after 6 months of training. We observed significant increase in values of immune system parameters and significant reduction in values of systemic inflammation markers after 6 months of both aerobic and resistance training in addition there were significant differences between both types of exercise training where aerobic exercise gained more remarkable effects.

The results of this study showed that after six months, the number of lymphocytes cells (CD3, CD4 and CD8) more significantly increased and CD4/CD8 ratio more significantly decreased in group(A) taking aerobic exercises as compared to group(B) taking resisted exercises. Cell numbers are expected to decrease due to aging process. This finding is consistent with other studies, while other contradicting studies have made different observations. As our results agreed with Peeri and colleagues who enrolled 40 healthy aged males in a 6 months aerobic exercise training program and noticed that the number of CD4 and CD8 cells significantly increased after aerobic exercise training along with increased values of VO_2max_^39^.

Although some research on aerobic exercise training has suggested improvements in the immune system for elderly subjects^40^–^42^. There are three randomized prospective trials of exercise and immune function that have been conducted in previously sedentary elderly humans^43^–^45^. Unfortunately, these studies have included small subject numbers and these were followed up over a short duration (usually 3 months or less). While, Crist et al. found that basal natural killer cell function was 33% higher in seven women who engaged in a 16 week aerobic exercise training program at 50% of heart rate reserve when compared to seven women who did not. However, this finding is difficult to interpret because pre-intervention measures were not taken^43^, where Nieman et al. found that a 3 month moderate aerobic exercise program (60% of heart rate reserve) failed to significantly increase natural killer cell cytotoxicity, T lymphocyte mitogenesis, natural killer cell or T cell subsets in previously sedentary women. These authors suggested that this 3 month period may have been too brief to significantly alter immune function^44^. In addition, Woods et al. also didn't find changes in the cellular immunity parameters such as lymphoproliferative responses and NK cell activity of elderly subjects undergoing 6 months of moderate aerobic exercise training^45^. Kapasi et al. had a small but significant increase in CD8^+^ (5%) cells in frail, elderly, nursing home residents after 8 weeks of an aerobic and resistance exercise program that was subsequently lost when evaluated at 32 weeks^46^.

On the other hand, the effect of resistance training on immune function in the elderly has been investigated in a limited number of studies. Most of them found that 8–12 weeks of resistance training programs had minimal effects on resting inflammatory, innate, or acquired immune parameters, as assessed by analysis of peripheral blood^47^–^50^. However, Raso et al. proved that a 12-month moderate resistance training program increases muscle strength, but it does not change immune phenotypic and functional parameters of 42 healthy sedentary elderly women^51^. Also, McFarlin et al. have shown that resting natural killer cells increases as a result of a vigorous resistance training program in elderly women^52^. Moreover, Miles et al. investigated different intensities of resistance training in young women, and concluded that anaerobic intensity is associated with increased strength and workload but not with changes in T cell proliferation responses^53^. This is also consistent with studies that have shown a lack of improvement in immune function with high-intensity exercises^54^,^55^. Moreover, Rall et al. found that 3 months of progressive resistance strength training that resulted in a 36% increase in strength did not induce changes in leukocyte subsets, cytokine production, lymphocyte proliferation or the delayed-type hypersensitivity response to a battery of antigens^56^.

The mechanism of immune system improvement due to physical activities is not clearly understood, but one of the factors considered is the increase in free radicals production^57^,^58^. Oxygen consumption increases up to 10 folds during exercise and so the number of free radicals increases dramatically. Thus, the immune system acquires more capacity to combat harmful free radicals available in blood, production of anti-oxidant enzymes such as superoxide dismutase, catalase and glutathione peroxides increases, this process leads to the adjustment of antioxidant enzymes performances, cell-mediated immune response and increase in the numbers of CD4 and CD8 cells^57^. Decrease in sympathetic system performance and β adrenergic receptors sensitivity due to aging may be compensated by moderate exercise and increase in secretion of catecholamine and stimulation of spleen^59^, lymphatic nodes, and thymus and lymphatic cells with proliferation of T-cells and CD4 and CD8. Stimulation of β adrenergic receptors may result in CAMP activation and production of lymphocytes^60^,^61^. Positive changes in Th1 to Th2 ratio has also been mentioned as an effective response due to sport activities^62^.

Our results demonstrate that both aerobic and resistance exercise training causes a decrease in TNF-α, IL-6 and CRP levels, in addition to increase in IL-10 level which suggests that exercise training can reduce inflammation in elderly individuals with more significant changes following aerobic exercise training. Several studies have shown that moderate physical exercise promotes the modulation of inflammation^63^–^65^. Several large cohort studies have found a relationship between self-reported physical activity levels and systemic markers of inflammation: higher levels of physical activity are coupled to lower levels of circulating inflammatory markers in elderly individuals^66^–^68^. Regarding the aerobic exercise training, our results agreed with Nicklas et al., who showed that regular aerobic exercise training was efficient in lowering IL-6 levels even without weight loss^69^. Also, Santos and colleagues had twenty-two male, sedentary, healthy, elderly volunteers perform moderate aerobic exercise training for 60 min/day, 3 days/week for 24 weeks and concluded that 6 months of aerobic exercise training can improve sleep in the elderly via anti-inflammatory effect of aerobic training which modifies cytokine profiles (reduced IL-6 and TNF-α and increased IL-10)^70^. However, Kohut et al. reported that 10-months of aerobic, but not resistance exercise, significantly reduces serum inflammatory mediators in older adults^71^. In addition, Bote et al. demonstrated that 8-months (2 sessions/week, 60-min/session) of aquatic-based exercise training tempered neutrophil activation (chemotaxis) and decreased systemic levels of IL-8 and noradrenalin compared to controls^72^. On the other hand, our results regarding resistance exercise training agreed with White et al., who found alterations in the biomarkers of inflammation after 8 weeks of resistance training in individuals with multiple sclerosis^73^. Where, Prestes et al.performed resistance training for 16 weeks in elderly sedentary and found reductions in the levels of IL-6 after training^74^. Moreover, our results confirmed that aerobic exercise training is more appropriate to modify the inflammatory markers among elderly and this agreed with Ploeger et al. who reported that moderate aerobic exercise training has been recommended as an anti-inflammatory therapy^75^.

The three possible mechanisms of exercise anti-inflammatory effects include reduction in visceral fat mass^76^; reduction in the circulating numbers of pro-inflammatory monocytes^77^ and an increase in the circulating numbers of regulatory T cells^78^. Moreover, Hong and colleagues show that cardiorespiratory fitness is associated with reduced low grade inflammation which may in part be mediated by enhancing the ability of immune cells to suppress inflammatory responses via adrenergic receptors^79^.

The present study has important strengths and limitations. The major strength is the supervised nature of the study. Supervising physical activity removes the need to question compliance or to rely on food and activity questionnaires. Further, all exercise sessions were supervised and adherence to the activities was essentially 100%. Moreover, the study was randomized; hence, we can extrapolate adherence to the general population. On the other hand, the major limitations is only elderly subjects where enrolled in the study, so the value of this study only related to women in this age group, in addition, a small sample size in both groups may limit the possibility of generalization of the findings in the present study. Finally, within the limit of this study, aerobic exercise is more appropriate in modulating immune system and inflammatory markers among the elderly population. Further researches are needed to explore the impact of weight reduction on quality of life and other biochemical parameters among elderly subjects.

## Conclusion

The current study provides evidence that aerobic exercise is more appropriate in modulatong immune system and inflammatory markers and among elderly population.

## References

[1] Pawelec G (2006). Immunity and ageing in man. Exp Gerontol.

[2] Koch S, Larbi A, Ozcelik D, Solana R, Gouttefangeas C, Attig S, Wikby A, Strindhall J, Franceschi C, Pawelec G (2007). Cytomegalovirus infection: a driving force in human T cell immunosenescence. Ann NY Acad Sci.

[3] Franceschi C, Capri M, Monti D, Giunta S, Olivieri F, Sevini F, Panourgia MP, Invidia L, Celani L, Scurti M, Cevenini E, Castellani GC, Salvioli S (2007). Inflammaging and anti-inflammaging: a systemic perspective on aging and longevity emerged from studies in humans. Mech Ageing Dev.

[4] Bruunsgaard H, Skinhoj P, Pedersen AN, Schroll M, Pedersen BK (2000). Ageing, tumour necrosis factor-alpha (TNF-alpha) and atherosclerosis. Clin Exp Immunol.

[5] Toth MJ, Ades PA, Tischler MD, Tracy RP, LeWinter MM (2006). Immune activation is associated with reduced skeletal muscle mass and physical function in chronic heart failure. Int J Cardiol.

[6] Allin KH, Nordestgaard BG, Zacho J, Tybjaerg-Hansen A, Bojesen SE (2010). Creactive protein and the risk ofcancer: a mendelian randomization study. J Natl Cancer Inst.

[7] Wensley F, Gao P, Burgess S, Kaptoge S, Di AE, Shah T, Engert JC, Clarke R, Davey-Smith G, Nordestgaard BG, Saleheen D, Samani NJ, Sandhu M, Anand S, Pepys MB, Smeeth L, Whittaker J, Casas JP, Thompson SG, Hingorani AD, Danesh J (2011). Association between C reactive protein and coronary heart disease: mendelian randomisation analysis based on individual participant data. BMJ.

[8] Goh J, Ladiges W (2014). Exercise enhances wound healing and prevents cancer progression during aging by targeting macrophage polarity. Mechanisms of Ageing and Development.

[9] Woods JA, Ceddia MA, Wolters BW (1999). Effects of 6 months of moderate aerobic exercise training on immune function in the elderly. Mechanisms of Ageing and Development.

[10] Fairey AS, Courneya KS, Field CJ (2005). Randomized controlled trial of exercise and blood immune function in postmenopausal breast cancer survivors. Journal of Applied Physiology.

[11] Woods JA, Lowder TW, Keylock KT (2002). Can exercise training improve immune function in the aged?. Ann N Y Acad Sci.

[12] Kohut ML, Senchina DS (2004). Reversing age-associated immunosenescence via exercise. Exerc Immunol Rev.

[13] Bruunsgaard H (2005). Physical activity and modulation of systemic low-level inflammation. J Leukoc Biol.

[14] Balducci S, Zanuso S, Nicolucci A, Fernando F, Cavallo S, Cardelli P (2010). Anti-inflammatory effect of exercise training in subjects with type 2 diabetes and the metabolic syndrome is dependent on exercise modalities and independent of weight loss. Nutrition Metabolism and Cardiovascular Diseases.

[15] Nishida Y, Higaki Y, Taguchi N, Hara M, Nakamura K, Nanri H (2014). Objectively measured physical activity and inflammatory cytokine levels in middle-aged Japanese people. Preventive Medicine.

[16] Palmefors H, DuttaRoy S, Rundqvist B, Borjesson M (2014). The effect of physical activity or exercise on key biomarkers in atherosclerosis—a systematic review. Atherosclerosis.

[17] Kapasi ZF, Ouslander JG, Schnelle JF, Kutner M, Fahey JL (2003). Effects of an exercise intervention on immunologic parameters in frail elderly nursing home residents. J Gerontol Med Sci.

[18] Woods JA, Ceddia MA, Wolters BW, Evan JK, Lu Q, Mcauley E (1999). Effects of 6 months of moderate aerobic exercise training on immune function in the elderly. Mech Ageing Dev.

[19] Ploeger HE, Takken T, de Greef MH, Timmons BW (2009). The effects of acute and chronic exercise on inflammatory markers in children and adults with a chronic inflammatory disease: a systematic review. Exerc Immunol Rev.

[20] Prestes J, Shiguemoto G, Botero JP (2009). Effects of resistance training on resistin, leptin, cytokines, and muscle force in elderly post-menopausal women. J Sports Sci.

[21] White LJ, Castellano V, McCoY SC (2006). Cytokine responses to resistance training in people with multiple sclerosis. J Sports Sci.

[22] Bruunsgaard H, Pedersen BK (2000). Special feature for the olympics: effects of exercise on the immune system in the elderly population. Immunol Cell Biol.

[23] Kohut ML, Senchina DS (2004). Reversing age-associated immunosenescence via exercise. Exerc Immunol Rev.

[24] Woods JA, Lowder TW, Keylock KT (2002). Can exercise training improve immune function in the aged?. Ann N Y Acad Sci.

[25] Drela N, Kozdron E, Szczypiorski P (2004). Moderate exercise may attenuate some aspects of immunosenescence. BMC Geriatr.

[26] Nieman DC, Cook VD, Henson DA (1995). Moderate exercise training and natural killer cell cytotoxic activity in breast cancer patients. Int J Sports Med.

[27] Shinkai S, Kohno H, Kimura K (1995). Physical activity and immune senescence in men. Med Sci Sports Exerc.

[28] Mcfarlin BK, Flynn MG, Campbell WW, Stewart LK, Timmerman KL (2004). TLR4 is lower in resistance-trained older women and related to inflammatory cytokines. Med Sci Sports Exerc.

[29] Kapasi ZF, Ouslander JG, Schnelle JF, Kutner M, Fahey JL (2003). Effects of an exercise intervention on immunologic parameters in frail elderly nursing home residents. J Gerontol Med Sci.

[30] Rall LC, Roubenoff R, Cannon JG, Abad LW, Dinarello CA, Meydani SN (1996). Effects of progressive resistance training on immune response in aging and chronic inflammation. Med Sci Sport Exerc.

[31] Robergs RA, Landwehr R (2002). The surprising history of the “HRmax=220-age” equation. J Exerc Physiol Online.

[32] Ramalho AC, de Lourdes Lima M, Nunes F, Cambuí Z, Barbosa C, Andrade A, Viana A, Martins M, Abrantes V, Aragão C, Temístocles M (2006). The effect of resistance versus aerobic training on metabolic control in patients with type-1 diabetes mellitus. Diabetes Res Clin Pract.

[33] Hakim FT, Gress RE (2005). Immunosenescence: immune deficits in the elderly and therapeutic strategies to enhance immune competence. Exp Rev Clin Immunol.

[34] Bruunsgaard H, Pedersen BK (2000). Special feature for the olympics: effects of exercise on the immune system in the elderly population. Immunol Cell Biol.

[35] Drela N, Kozdron E, Szczypiorski P (2004). Moderate exercise may attenuate some aspects of immunosenescence. BMC Geriatr.

[36] Rall LC, Roubenoff R, Cannon JG, Abad LW, Dinarello CA, Meydani SN (1996). Effects of progressive resistance training on immune response in aging and chronic inflammation. Med Sci Sports Exerc.

[37] Kohut ML, Senchina DS (2004). Reversing age-associated immunosenescence via exercise. Exerc Immunol Rev.

[38] Woods JA, Lowder TW, Keylock KT (2002). Can exercise training improve immune function in the aged?. Ann N Y Acad Sci.

[39] Peeri M, Azarbayjani M, Akbarpour M, Ebrahimi M (2011). The effect of aerobic training on the immune system of aging men. Annals of Biological Research.

[40] Drela N, Kozdron E, Szczypiorski P (2004). Moderate exercise may attenuate some aspects of immunosenescence. BMC Geriatr.

[41] Nieman DC, Cook VD, Henson DA (1995). Moderate exercise training and natural killer cell cytotoxic activity in breast cancer patients. Int J Sports Med.

[42] Shinkai S, Kohno H, Kimura K (1995). Physical activity and immune senescence in men. Med Sci Sports Exerc.

[43] Crist DM, Mackinnon LT, Thomson RF, Atterbom HA, Egan PA (1989). Physical exercise increases natural cellular-mediated tumor cytotoxicity in the elderly women. Gerontology.

[44] Nieman DC, Henson DA, Gusewitch G (1993). Physical activity and immune function in elderly women. Med Sci Sports Exerc.

[45] Woods JA, Ceddia MA, Wolters BW, Evan JK, Lu Q, Mcauley E (1999). Effects of 6 months of moderate aerobic exercise training on immune function in the elderly. Mech Ageing Dev.

[46] Kapasi ZF, Ouslander JG, Schnelle JF, Kutner M, Fahey JL (2003). Effects of an exercise intervention on immunologic parameters in frail elderly nursing home residents. J Gerontol Med Sci.

[47] Bermon S, Philip P, Candito M, Ferrari P, Dolisi C (2001). Effects of strength exercise and training on the natural killer cell counts in elderly humans. J Sports Med Phys Fitness.

[48] Flynn MG, Fahlman M, Braun WA (1999). Effects of resistance training on selected indexes of immune function in elderly women. J Appl Physiol.

[49] Flynn MG, Mcfarlin BKMD, Phillips MD, Stewart LK, Timmerman KL (2003). Toll-like receptor 4 and CD14 mRNA expression are lower in resistive exercise-trained elderly women. J Appl Physiol.

[50] Rall LC, Roubenoff R, Cannon JG, Abad LW, Dinarello CA, Meydani SN (1996). Effects of progressive resistance training on immune response in aging and chronic inflammation. Med Sci Sports Exerc.

[51] Raso V, Benard G, DA Silva Duarte AJ, Natale VM (2007). Effect of Resistance Training on Immunological Parameters of Healthy Elderly Women. Med Sci Sports Exerc.

[52] Mcfarlin BK, Flynn MG, Campbell WW, Stewart LK, Timmerman KL (2004). TLR4 is lower in resistance-trained older women and related to inflammatory cytokines. Med Sci Sports Exerc.

[53] Miles MP, Kraemer WJ, Nindl BC (2003). Strength, workload, anaerobic intensity and the immune response to resistance exercise in women. Acta Physiol Scand.

[54] Bruunsgaard H, Pedersen BK (2000). Special feature for the olympics: effects of exercise on the immune system in the elderly population. Immunol Cell Biol.

[55] Woods JA, Lowder TW, Keylock KT (2002). Can exercise training improve immune function in the aged?. Ann N Y Acad Sci.

[56] Rall LC, Roubenoff R, Cannon JG, Abad LW, Dinarello CA, Meydani SN (1996). Effects of progressive resistance training on immune response in aging and chronic inflammation. Med Sci Sport Exerc.

[57] Finaud J, Lac G, Filaire E (2006). Oxidative stress : relationship with exercise and training. Sports Med.

[58] Baumann CA, Badamchian M, Goldstein AL (1997). Thymosin alpha 1 antagonizes dexamethasone and CD3-induced apoptosis of CD4^+^ CD8^+^ thymocytes through the activation of cAMP and protein kinase C dependent second messenger pathways. Mech Ageing Dev.

[59] Bouissou P, Guezennec CY, Galen FX, Defer G, Fiet J, Pesquies PC (1988). Dissociated response of aldosterone from plasma renin activity during prolonged exercise under hypoxia. Horm Metab Res.

[60] Stock C, Schaller K, Baum M, Liesen H, Weiss M (1995). Catecholamines, lymphocyte subsets, and cyclic adenosine monophosphate production in mononuclear cells and CD4^+^ cells in response to submaximal resistance exercise. Eur J Appl Physiol Occup Physiol.

[61] Saha B, Mondal AC, Majumder J, Basu S, Dasgupta PS (2001). Physiological concentrations of dopamine inhibit the proliferation and cytotoxicity of human CD4^+^ and CD8^+^ T cells in vitro: a receptor-mediated mechanism. Neuroimmuno modulation.

[62] Shearer GM (1997). Th1/Th2 changes in aging. Mech Ageing Dev.

[63] Donges CE, Duffield R, Drinkwater EJ (2010). Effects of resistance or aerobic exercise training on interleukin-6, c-reactive protein, and body composition. Med Sci Sports Exerc.

[64] Balducci S, Zanuso S, Nicolucci A (2010). Anti-inflammatory effect of exercise training in subjects with type 2 diabetes and the metabolic syndrome is dependent on exercise modalities and independent of weight loss. Nutr Metab Cardiovasc Dis.

[65] Libardi CA, Souza GV, Cavaglieri CR (2012). Effect of resistance, endurance, and concurrent training on TNF-a, IL-6, and CRP. Med Sci Sports Exerc.

[66] Geffken DF, Cushman M, Burke GL, Polak JF, Sakkinen PA, Tracy RP (2001). Association between physical activity and markers of inflammation in a healthy elderly population. Am J Epidemiol.

[67] Colbert LH, Visser M, Simonsick EM, Tracy RP, Newman AB, Kritchevsky SB, Pahor M, Taaffe DR, Brach J, Rubin S, Harris TB (2004). Physical activity, exercise, and inflammatory markers in older adults: findings from the Health, Aging and Body Composition Study. J Am Geriatr Soc.

[68] Yu Z, Ye X, Wang J, Qi Q, Franco OH, Rennie KL, Pan A, Li H, Liu Y, Hu FB, Lin X (2009). Associations of physical activity with inflammatory factors, adipocytokines, and metabolic syndrome in middle-aged and older chinese people. Circulation.

[69] Nicklas BJ, Hsu FC, Brinkley TJ, Church T, Goodpaster BH, Kritchevsky SB, Pahor M (2008). Exercise training and plasma C-reactive protein and interleukin- 6 in elderly people. J Am Geriatr Soc.

[70] Santos R, Viana V, Boscolo R, Marques V, Santana M, Lira F, Tufik S, de Mello M (2012). Moderate exercise training modulates cytokine profile and sleep in elderly people. Cytokine.

[71] Kohut ML, McCann DA, Russell DW, Konopka DN, Cunnick JE, Franke WD, Vanderah E (2006). Aerobic exercise, but not flexibility/resistance exercise, reduces serum IL-18, CRP, and IL-6 independent of beta-blockers, BMI, and psychosocial factors in older adults. Brain Behav Immun.

[72] Bote ME, Garcia JJ, Hinchado MD, Ortega E (2014). An exploratory study of the effect of regular aquatic exercise on the function of neutrophils from women with fibromyalgia: role of IL-8 and noradrenaline. Brain Behav Brain Behav Immun.

[73] White LJ, Castellano V, McCoY SC (2006). Cytokine responses to resistance training in people with multiple sclerosis. J Sports Sci.

[74] Prestes J, Shiguemoto G, Botero JP (2009). Effects of resistance training on resistin, leptin, cytokines, and muscle force in elderly post-menopausal women. J Sports Sci.

[75] Ploeger HE, Takken T, de Greef MH, Timmons BW (2009). The effects of acute and chronic exercise on inflammatory markers in children and adults with a chronic inflammatory disease: a systematic review. Exerc Immunol Rev.

[76] Mathur M, Pedersen B (2008). Exercise as a mean to control low-grade inflammation. Mediators Inflamm.

[77] Timmerman K, Flynn M, Coen P, Markofski M, Pence B (2008). Exercise training-induced lowering of inflammatory (CD14+CD16+) monocytes: a role in the anti-inflammatory influence of exercise?. Leukoc Biol.

[78] Wang J, Song H, Tang X, Yang Y, Vieira VJ, Niu Y, Ma Y (2012). Effect of exercise training intensity on murine T regulatory cells and vaccination response. Scand J Med Sci Sports.

[79] Hong S, Dimitrov S, Pruitt C, Shaikh F, Beg N (2014). Benefit of physical fitness against inflammation in obesity: role of beta adrenergic receptors. Brain Behav Immun.

